# Study on mechanism of Spatholobi Caulis in the treatment of the hand-foot skin reaction induced by targeted drug therapy based on network pharmacology and molecular docking: An observational study

**DOI:** 10.1097/MD.0000000000041085

**Published:** 2025-01-10

**Authors:** Quan-yao Li, Huan-nan Meng, Li-qiu Yao, Hui Liu, He-xin Tan, Dan Lin, Jun Shi

**Affiliations:** a Department of Traditional Chinese Medicine, Shanghai Fourth People’s Hospital Affiliated to Tongji University of Medicine, Shanghai, China; b Department of oncology, Shanghai Municipal Hospital of Traditional Chinese Medicine, Shanghai University of Traditional Chinese Medicine, Shanghai, China; c Department of Traditional Chinese Medicine, Jing’an District Hospital of Traditional Chinese Medicine, Shanghai, China; d Department of oncology, Wuxi NO. 2 Chinese Medicine Hospital, Jiangsu, China; e Department of Chinese medicine identification, Naval Medical University, Shanghai, China; f Department of oncology, Yueyang Hospital of Integrated Traditional Chinese and Western Medicine Affiliated to Shanghai University of Traditional Chinese Medicine, Shanghai, China.

**Keywords:** hand-foot skin reaction, molecular docking, network pharmacology, Spatholobi Caulis, traditional Chinese medicine

## Abstract

Based on network pharmacology and molecular docking methods, this study explored its active compounds and confirmed its potential mechanism of action against Hand-foot skin reaction induced by tumor-targeted drugs. Traditional Chinese Medicine Systems Pharmacology Database and Analysis Platform and UniProt Database were used to obtain the active ingredients and target proteins of Spatholobi Caulis. All hand-foot skin reaction (HFSR)-related targets were obtained with the help of the Human Gene Database, Online Mendelian Inheritance in Humans (OMIM), DisGeNET and DrugBank databases. Cytoscape 3.7.1 software was used to construct the active ingredient-target visualization network of Spatholobi Caulis, and the common target network of Spatholobi Caulis and HFSR. And through the BisoGenet plug-in in Cytoscape, PPI network topology analysis was performed. The Metescape database and online mapping tool platform were used for Gene Ontology (GO) function and Kyoto Encyclopedia of Genes Genomes (KEGG) pathway enrichment analysis to identify the key pathways of Spatholobi Caulis. Finally, Autodock Vina software and PyMol 2.5 software were used for molecular docking verification. There were 24 active components of Spatholobi Caulis and 244 target genes, 1635 disease-related target genes, 51 common target genes of Spatholobi Caulis and Hand-foot skin reaction, and key targets included NTRK1, epidermal growth factor receptor (EGFR), APP, TP53, HSP90AB1, HSP90AA, CUL3, etc. GO functional analysis involved a total of 66 molecular functions, 39 cellular components, and 817 biological processes. KEGG pathway analysis found 154 related signaling pathways, mainly enriched in Pathways in cancer, Human cytomegalovirus infection, Kaposi arcoma-associated herpesvirus infection, panic cancer, EGFR tyrosine kinase inhibitor resistance, P13K-Akt signaling pathway, Proteoglycans in cancer, HIF-1 signaling pathway and Ras signaling pathway, etc. Molecular docking results showed that luteolin, the active component of Spatholobi Caulis, had a high affinity with EGFR. Medicagol, the active components of Spatholobi Caulis, is proved in the Hand-foot skin reaction induced by lung cancer targeted therapy by regulating multiple signaling pathways through EGFR. It is confirmed that the treatment of Hand-foot skin reaction has the characteristics of multi-component, multi-target and multi-pathway regulation.

## 1. Introduction

In recent years, with the increasing incidence of malignant tumors, they have become the primary lethal factor for human beings. As an important part of the treatment of malignant tumors, targeted drug therapy is a therapeutic method that targets the identified carcinogenic sites at the cellular and molecular level and is designed to corresponding therapeutic drugs, which specifically bind to them and cause-specific death of tumor cells.^[1]^ Targeted drug therapy has become the first choice for a variety of malignant tumors because of its significant clinical efficacy and good tolerability, which brings hope to patients with malignant tumors. However, targeted drug therapy can cause a series of adverse skin reactions, among which Hand-foot skin reaction is the most common.^[2,3]^ Hand-foot skin reaction, also known as palmoplantar sensory loss erythema, Burgdof’s syndrome. The clinical manifestations are swelling, erythema, scaling, chapped, pustules, itching, pain, and other paresthesias on the hands and feet,^[4]^ which seriously affect the quality of life of patients. The incidence of hand-foot skin reaction (HFSR) is relatively high, and studies have confirmed that the incidence can be as high as 60.5%,^[5]^ and the incidence is closely related to the type and the dosage of the targeted drug.^[6]^ The data shows that the incidence of HFSR increases significantly when anti-vascular endothelial growth factor antibodies are combined with multi-kinase inhibitors,^[7]^ which seriously affects the compliance and tolerance of patients.

At present, the clinical treatment is mainly through oral vitamins, COX-2 inhibitors, adjustment of the dosage and other symptomatic treatment,^[8]^ but the efficacy is not good with a high recurrence rate. In severe cases, patients may experience medication interruption or even termination of treatment, which seriously affects the anti-tumor efficacy and the quality of life of patients.^[9,10]^ Therefore, it is of great significance to actively seek an effective method. Traditional Chinese medicine has unique advantages in the treatment of hand-foot skin reaction, but neither acupuncture nor traditional Chinese medicine has the support of clinical big data research as the support point, and it is difficult to standardize the implementation. Therefore, how to apply traditional Chinese medicine theory to the prevention and treatment of HFSR in a more targeted and effective manner is an urgent problem to be solved at present.

HFSR is mainly characterized by redness, swelling, heat and pain, and it belongs to the category of “blood arthralgia” in traditional Chinese medicine. “Huangdi Neijing” records the origin of blood paralysis. “Su Wen” and “Ling Shu” are parts of the “Huangdi Neijing,” “Su Wen” records: “Blood coagulation on the skin, it is paralysis,” and “Ling Shu” records: “When evil enters the yin, it is blood paralysis.” Therefore, it can be considered that blood paralysis is located in the limbs and is closely related to the liver, spleen, lung, kidney, etc. Traditional Chinese medicine believes that targeted drugs are “heat poison,”^[11]^ which can easily consume and damage the healthy qi and blood of the human body. In addition, tumor patients have a deficiency of the body and lack of qi and blood, resulting in a deficiency of qi and blood, stasis of blood vessels, and dystrophy of meridians. Therefore, the basic principle in the treatment should be “promoting blood circulation, nourishing blood, dredging collaterals and relieving pain.”

Spatholobi Caulis is the dry cane of the legume Spatholobus suberectus Dunn, which is mainly composed of xylem and phloem.^[12]^ It is called blood vine because of the red liquid that leaks out when the fresh rhizome is cut off. As a natural Chinese herbal medicine with the same origin as medicine and food, it is sweet and bitter in taste and warm in nature, in “Bencao Gangmu Shiyi,” it was recorded that “its vine is the most activating blood, warming the waist and knees that are already cold,” which has the effects of promoting blood circulation, nourishing blood, dredging collaterals and relieving pain. Modern pharmacological studies have shown that Spatholobi Caulis has the functions of regulating the immune system, improving hematopoietic function,^[13]^ bidirectional regulation of anticoagulation, pro-coagulation, anti-fibrinolysis and pro-fibrinolysis, and anti-tumor effects.^[14]^ In recent years, it is often used in the treatment of non-small cell lung cancer with targeted drug therapy that causes adverse skin reactions, which can effectively improve the clinical symptoms of patients and improve their quality of life. Studies have found that the flavonoids of the effective extract of Spatholobi Caulis are the key substances to exert anti-cancer effects^[15]^ and reduce local skin damage.^[16]^ However, the efficacy and molecular mechanism of Spatholobi Caulis in the treatment of HFSR are not yet clear. Therefore, based on the network pharmacology and molecular docking, this study predicted the potential components, targets and pathways of Spatholobi Caulis in the treatment of HFSR, and confirmed its potential mechanism of Spatholobi Caulis against HFSR induced by tumor-targeted drugs. The purpose is to provide a scientific basis for the clinical application and further research of Spatholobi Caulis in the treatment of HFSR.

## 2. Materials and methods

### 2.1. Screening of the active ingredients and action targets of Spatholobi Caulis

The chemical constituents of Spatholobi Caulis were retrieved through the Traditional Chinese Medicine Systems Pharmacology Database and Analysis Platform^[17]^ (TCMSP, https://www.tcmsp-e.com/). Oral bioavailability (OB) ≥ 30% and drug likeness (DL) ≥ 0.18 are the conditions to screen active ingredients. The TCMSP platform was used to obtain the target proteins of the active ingredients, and the targets corresponding to the screened active chemical ingredients match the corresponding known human gene targets in the UniProt database^[18]^ (https://www.uniprot.org/) to obtain the target protein and gene of Spatholobi Caulis.

### 2.2. Getting HFSR targets

Using “Hand-foot skin reaction” as the key word, the Human Gene Database^[19]^ (https://www.genecards.org) and the online Mendelian Inheritance Database of Humans (http://www.omim.org), DisGeNET database^[20]^ (https://www.disgenet.org) and DrugBank database^[21]^ (https://go.drugbank.com) were searched, which all obtained targets related to HFSR.

### 2.3. Screening of common targets of drugs and diseases

The Venn online mapping software (http://www.bioinformatics.com.cn/) was used to obtain the common targets of the potential targets of Spatholobi Caulis and HFSR disease targets, and draw a Venn diagram. The common targets were imported into Cytoscape 3.7.1 software to obtain the common target network of Spatholobi Caulis and HFSR.

### 2.4. Building protein interaction networks

The potential targets of Spatholobi Caulis and HFSR were imported into the BisoGenet plug-in of Cytoscape 3.7.1 software, and the PPI network topology analysis was performed with degree, betweenness, closeness and LAC as parameters.

### 2.5. GO functional enrichment analysis and KEGG pathway enrichment analysis

The common targets of drugs and diseases were entered into the Metascape database^[22]^ (http://metascape.org), and the species was limited to “Homo sapiens.” The results were analyzed under the conditions of *P* < .01 and Enrichment > 1.5, and the top 20 Gene Ontology (GO) functional enrichment results and Kyoto Encyclopedia of Genes Genomes (KEGG) pathway enrichment results were selected. The GO functional enrichment analysis and KEGG pathway enrichment analysis were carried out using the online software mapping tool platform. The results were visualized and analyzed by bar graphs and bubble graphs to clarify the possible mechanism of action of Spatholobi Caulis in the treatment of HFSR.

### 2.6. Molecular docking

The crystal structure of TNFAIP3 protein used for molecular docking was downloaded from the PDB database, the ID of the PDB was 2rgp, and the 3D structure of the small molecule was downloaded from the TCMSP database,^[17]^ and the energy minimization was performed under the MMFF94 force field. AutoDock Vina 1.1.2 software was used for molecular docking work in this study. The receptor protein was processed with PyMol 2.5 before docking began, including the removal of water molecules, salt ions and small molecules. Subsequently, a cubic box with a side length of 22.5 angstroms was constructed with the centroid of the protein crystal ligand. In addition, ADFRsuite 1.0 was used to convert all processed small molecules and receptor proteins into PDBQT format required for AutoDock Vina 1.1.2 docking. When docking, the exhaustiveness of the global search is set to 32, and the rest of the parameters remain in the default settings. The highest-scoring docked conformation of the output was considered as the binding conformation and finally visualized using PyMol 2.5 docking results.

## 3. Results

### 3.1. Active components and targets of Spatholobi Caulis

**Figure 1.**
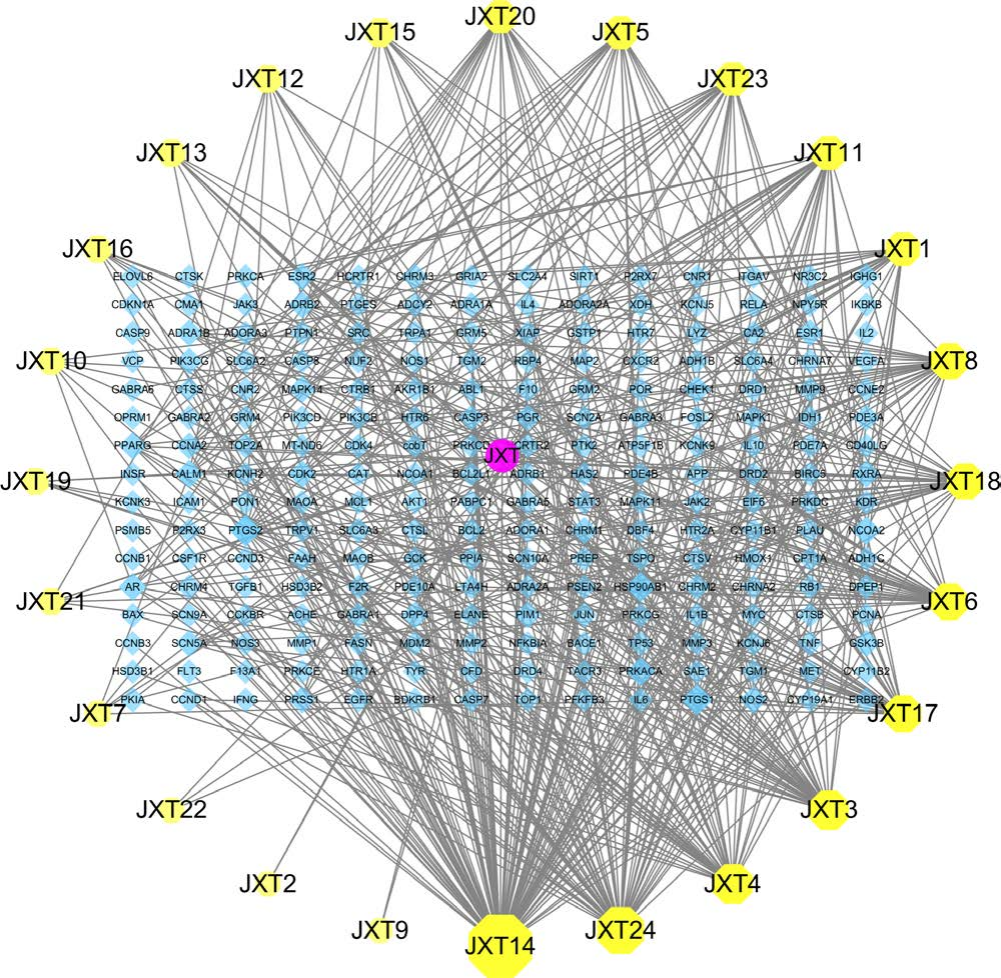
Active ingredient-potential target network diagram of Spatholobi Caulis.

The active ingredient-target network of Spatholobi Caulis was shown in the figure (Fig. 1), which includes a total of 249 nodes and 551 edges. The active components of Spatholobi Caulis were screened by TCMSP, and the results showed that there were a total of 24 active components (Table 1).

**Table 1:** Active ingredients of Spatholobi Caulis.

ID	MOL ID	Active ingredients	OB	DL
JXT1	MOL000296	Hederagenin	36.91	0.75
JXT2	MOL000033	(3S,8S,9S,10R,13R,14S,17R)-10,13-dimethyl-17-[(2R,5S)-5-propan-2-yloctan-2-yl]-2,3,4,7,8,9,11,12,14,15,16,17-dodecahydro-1H-cyclopenta[a]phenanthren-3-ol	36.23	0.78
JXT3	MOL000358	Beta-sitosterol	36.91	0.75
JXT4	MOL000392	Formononetin	69.67	0.21
JXT5	MOL000417	Calycosin	47.75	0.24
JXT6	MOL000449	Stigmasterol	43.83	0.76
JXT7	MOL000461	3,7-dihydroxy-6-methoxy-dihydroflavonol	43.8	0.26
JXT8	MOL000468	8-o-Methylreyusi	70.32	0.27
JXT9	MOL000469	3-Hydroxystigmast-5-en-7-one	40.93	0.78
JXT10	MOL000470	8-C-α-L-arabinosylluteolin	35.54	0.66
JXT11	MOL000471	Aloe-emodin	83.38	0.24
JXT12	MOL000483	(Z)-3-(4-hydroxy-3-methoxy-phenyl)-N-[2-(4-hydroxyphenyl)ethyl]acrylamide	118.35	0.26
JXT13	MOL000490	Petunidin	30.05	0.31
JXT14	MOL000491	Augelicin	37.5	0.66
JXT15	MOL000492	(+)-catechin	54.83	0.24
JXT16	MOL000493	Campesterol	37.58	0.71
JXT17	MOL000497	Licochalcone a	40.79	0.29
JXT18	MOL000500	Vestitol	74.66	0.21
JXT19	MOL000501	Consume close grain	68.12	0.27
JXT20	MOL000502	Cajinin	68.8	0.27
JXT21	MOL000503	Medicagol	57.49	0.6
JXT22	MOL000506	Lupinidine	61.89	0.21
JXT23	MOL000507	Psi-Baptigenin	70.12	0.31
JXT24	MOL000006	Luteolin	36.16	0.25

DL = drug likeness, OB = oral bioavailability.

### 3.2. Construction of correlation networks and PPI networks between Spatholobi Caulis and HFSR

**Figure 2.**
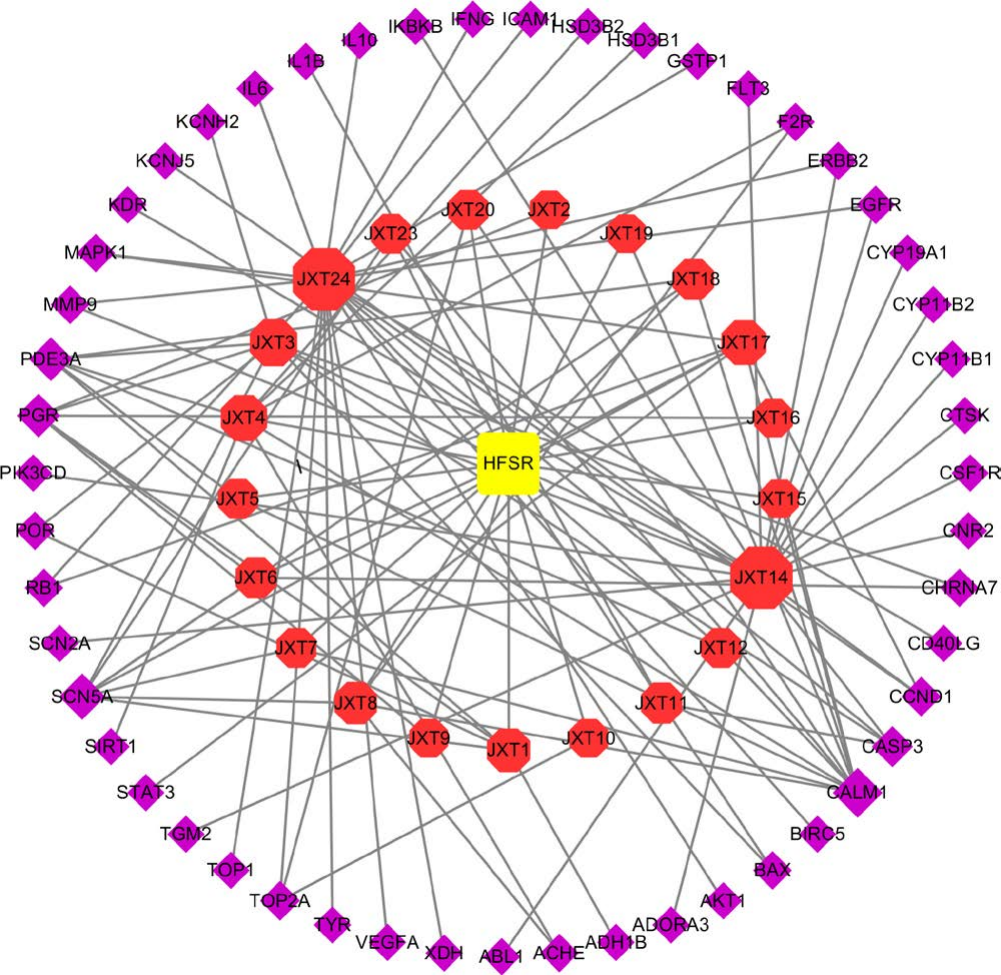
Network diagram of common target of Spatholobi Caulis and HFSR. HFSR = hand-foot skin reaction.

The effective chemical components were screened under the conditions of OB ≥ 30% and DL ≥ 0.18, and the corresponding target genes were detected by the UniProt database. Inputting the 224 drug target genes and 1635 disease target genes into the Venny online mapping software to obtain 51 common targets. The common targets were imported into Cytoscape 3.7.1 software to establish a network of common targets of Spatholobi Caulis and HFSR (Fig. 2).

**Figure 3.**
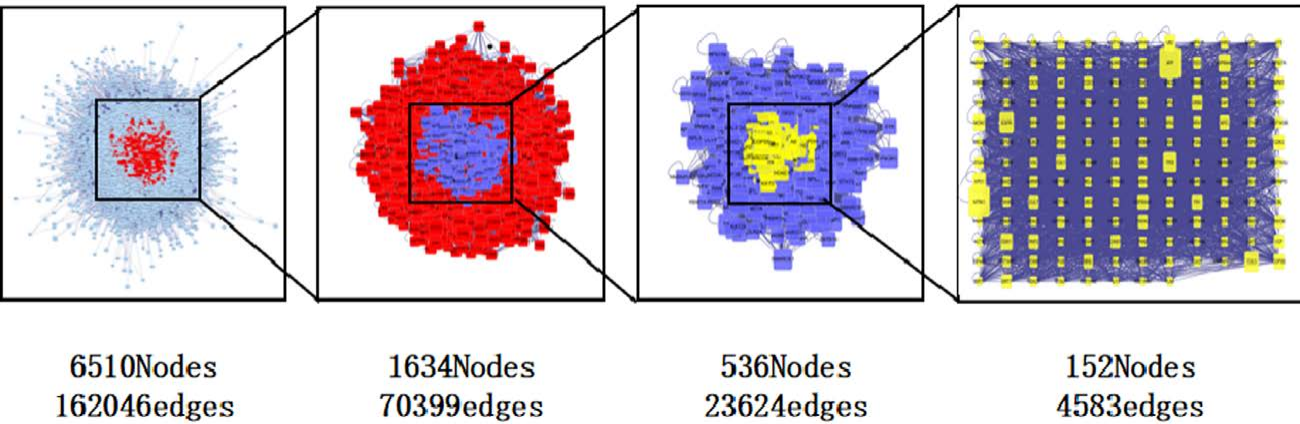
Screening of common target PPI.

The PPI network topology analysis was carried out on the potential targets of the treatment of HFSR by Spatholobi Caulis, which obtained a network with 6510 points and 162,046 edges. The Degree, Betweenness, Closeness and LAC values of each target in the network were calculated, and the subnetworks were extracted successively with the median as the threshold. First, extracting nodes with Degree ≥ 58, and obtaining a subnetwork with 1634 nodes and 70,399 edges. Secondly, extracting the nodes with Degree ≥ 95, Betweenness ≥ 615.523, Closeness ≥ 0.493 and LAC ≥ 13.774 from the subnetwork, and obtaining a subnetwork with 536 nodes and 23,624 edges. Finally, extracting the nodes with Degree ≥ 172, Betweenness ≥ 1932.843, Closeness ≥ 0.514 and LAC ≥ 25.009 from the obtained subnetwork, and constructing a subnetwork with 152 nodes and 4583 edges (Fig. 3). According to the PPI network topology analysis, the key targets were NTRK1, epidermal growth factor receptor (EGFR), APP, TP53, HSP90AB1, HSP90AA, and CUL3.

### 3.3. GO functional enrichment analysis

**Figure 4.**
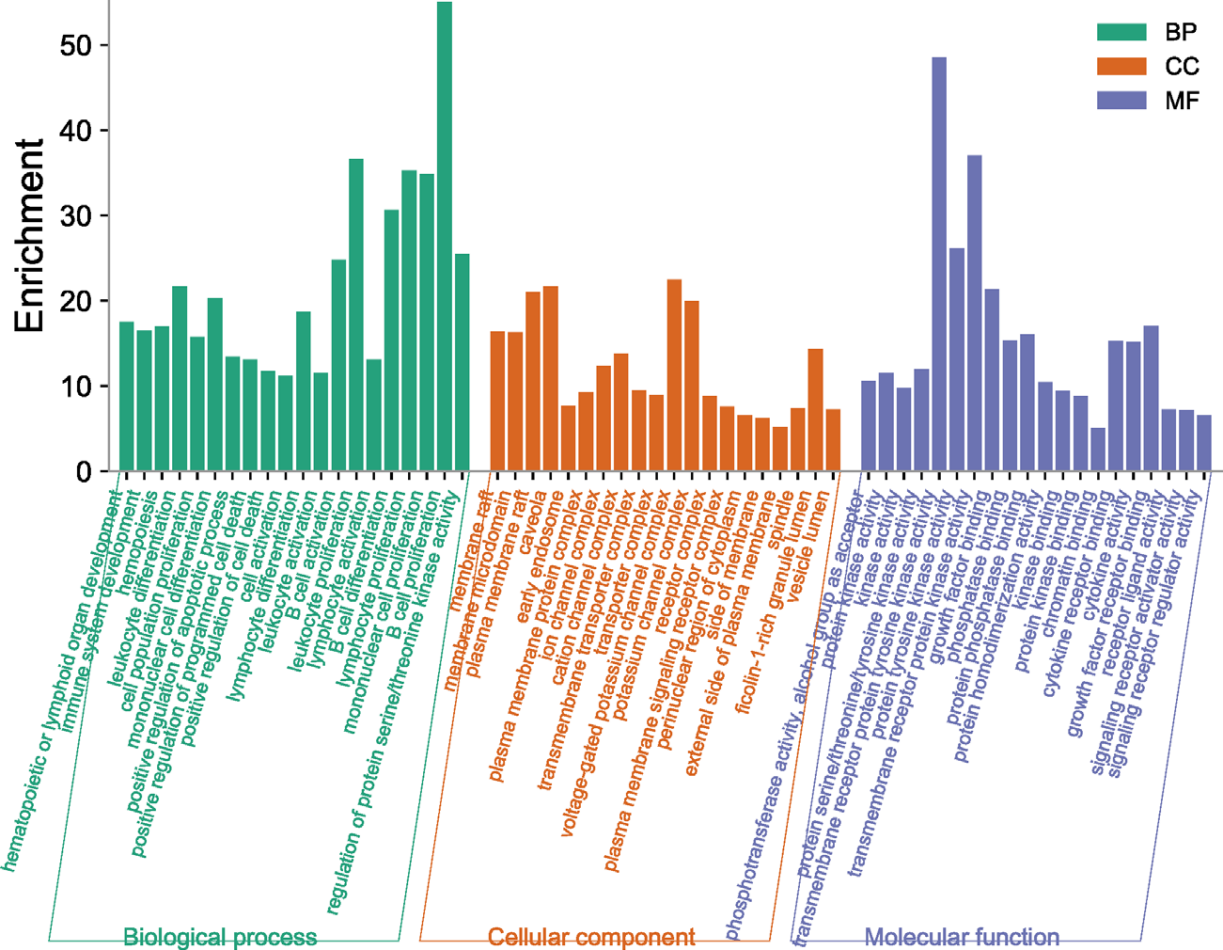
GO functional enrichment analysis. GO = Gene Ontology.

GO functional enrichment analysis revealed 66 molecular functions, 39 cellular components, and 817 biological processes. Visual analysis of the top 20 results of each branch. Molecular functions were mainly enriched in transmembrane receptor protein tyrosine kinase activity, protein tyrosine kinase activity and transmembrane receptor protein kinase activity, etc. The cellular components were mainly enriched in the plasma membrane, caveola, voltage-gated potassium channel complex and potassium channel complex, etc. Biological processes were mainly concentrated in leukocyte proliferation, lymphocyte proliferation, mononuclear cell proliferation and B cell proliferation, etc (Fig. 4).

### 3.4. KEGG pathway enrichment analysis

**Figure 5.**
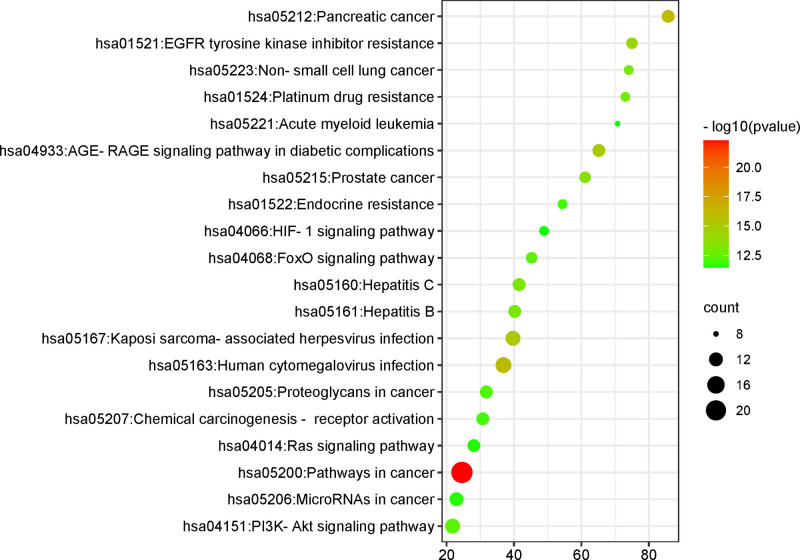
KEGG functional enrichment analysis. KEGG = Kyoto Encyclopedia of Genes Genomes.

KEGG pathway analysis obtained 154 related signaling pathways and visualized the top 20 results, which mainly involved pathways in cancer, Human cytomegalovirus infection, Kaposi arcoma-associated herpesvirus infection, panic cancer, EGFR tyrosine kinase inhibitor resistance, P13K-Akt signaling pathway, Proteoglycans in cancer, HIF-1 signaling pathway and Ras signaling pathway, etc (Fig. 5).

### 3.5. Molecular docking results between the active components of Spatholobi Caulis and EGFR protein

**Figure 6.**
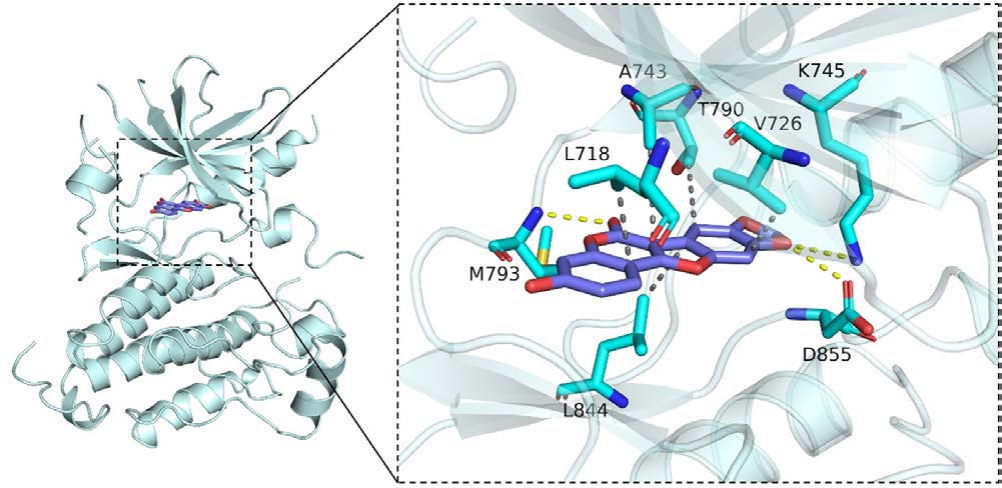
Molecular docking diagram of Medicagol and EGFR protein (The left image is the overall view, and the right is the partial view. The small molecule is shown as blue stick, amino acid side chains as light cyan cartoon, hydrogen bonding as yellow dotted line and hydrophobic interactions as gray dotted line). EGFR = epidermal growth factor receptor.

The results of the computer-simulated molecular docking experiment were seen in Table 2. The binding free energy of the 24 active ingredients of Spatholobi Caulis to the EGFR protein was all less than −5.5 kcal/mol, indicating that all the active ingredients of Spatholobi Caulis can bind to the EGFR protein molecule. The binding free energy of Medicagol (JXT21) and EGFR was the lowest (−9.2 kcal/mol), suggesting stronger bindings (Table 2). The small molecule Medicagol was tightly bound within the active pocket of the EGRR protein (Fig. 6). The oxygen atoms on the drug branch chain mainly formed strong hydrogen bonds with the phenolic hydroxyl groups at the ends of M793, D855, and K745 residues in EGFR. The benzene ring matched the hydrophobic region on the surface of the EGRR pocket and just fell into the groove consisting of L718, A743, T790, V726, L844 residues, forming a hydrophobic interaction.

**Table 2:** Free energy score of 24 active ingredients of Spatholobi Caulis combined with EGFR protein.

ID	CAS Rn	Chemical component	MW	EGFR docking score (kcal/mol)
JXT1	465-99-6	Hederagenin	472.71	−8.2
JXT2	10453-25-5	(3S,8S,9S,10R,13R,14S,17R)-10,13-dimethyl-17-[(2R,5S)-5-propan-2-yloctan-2-yl]-2,3,4,7,8,9,11,12,14,15,16,17-dodecahydro-1H-cyclopenta[a]phenanthren-3-ol	426.70	−8.9
JXT3	83-46-5	Beta-sitosterol	414.70	−8.7
JXT4	485-72-3	Formononetin	268.26	−8.3
JXT5	20575-57-9	Calycosin	284.26	−8.7
JXT6	83-48-7	Stigmasterol	412.70	−9
JXT7	34050-66-3	3,7-dihydroxy-6-methoxy-dihydroflavonol	286.28	−8.2
JXT8	37816-20-9	8-o-Methylreyusi	298.29	−8.3
JXT9	2034-74-4	3-Hydroxystigmast-5-en-7-one	428.70	−8.8
JXT10	-	8-C-α-L-arabinosylluteolin	418.3	−9.1
JXT11	481-72-1	Aloe-emodin	270.24	−8.7
JXT12	-	(Z)-3-(4-hydroxy-3-methoxy-phenyl)-N-[2-(4-hydroxyphenyl)ethyl]acrylamide	268.32	−8.9
JXT13	1429-30-7	Petunidin	352.70	−8.6
JXT14	523-50-2	Augelicin	186.16	−7.8
JXT15	154-23-4	(+)-catechin	290.27	−8.7
JXT16	474-62-4	Campesterol	400.70	−9.1
JXT17	58749-22-7	Licochalcone a	338.40	−8.5
JXT18	56701-24-7	Vestitol	272.29	−8.4
JXT19	–	Consume close grain	282.36	−8.6
JXT20	32884-36-9	Cajinin	300.26	−8.5
JXT21	1983-72-8	Medicagol	296.23	−9.2
JXT22	90-39-1	Lupinidine	234.38	−7.7
JXT23	90-29-9	Psi-Baptigenin	282.25	−9
JXT24	491-70-3	Luteolin	286.24	−9

CAS = chemical abstracts service, CAS Rn = CAS registry number, EGFR = epidermal growth factor receptor, MW = molecular weight.

## 4. Discussion

HFSR is a common skin toxicity in targeted drug therapy. A variety of targeted drugs such as apatinib and sorafenib can lead to the occurrence of HFSR, which is usually caused by abnormal epidermal homeostasis of keratinocytes.^[23]^ The main pathological manifestations are parakeratosis, dyskeratosis, perivascular lymphocyte infiltration in the dermis, and no specificity.^[24]^ However, the pathogenesis of HFSR has not been defined. The mainstream theories tend to be keratinocyte differentiation, vascular endothelial dysfunction, vascular repair, mechanical stress aggravating vascular damage, accumulation of drug concentrations in exocrine glands of hands and feet, and abnormal expression of tumor necrosis factor (TNF-α) and vascular endothelial growth factor (VEGF) and other mechanisms.^[25–27]^ This study found that there are 24 effective active ingredients in S. chinensis that play an important role in the treatment of HFSR, and involve a variety of target proteins and multiple signaling pathways. These results suggest that these active ingredients have potential research value.

A variety of active ingredients in Spatholobi Caulis have been shown to have therapeutic effects on the adverse reactions of hands and feet. Beta-sitosterol hydrogel formulations such as flavonoids could prolong skin residence time and showed a concentration-dependent antihyperalgesia effect in a rat inflammatory pain model.^[28]^ Formononetin enhanced Egr-1 transcription factor expression by activating ERK1/2 and p38 MAPK pathways, promoting endothelial repair and wound healing.^[29]^ Triterpenoids like hederagenin dose-dependently reduced iNOS and COX-2 protein levels and TNF-α, IL-1β and IL-6 mRNA levels in lipopolysaccharide-induced RAW 264.7 cell model, and inhibited hind paw skin thickness in mice increased, exerting anti-inflammatory and anti-edema effects.^[30]^ In summary, the effective and active components of Spatholobi Caulis could treat HFSR through a variety of signaling pathways and proteins.

KEGG enrichment analysis showed that 154 related signaling pathways were involved in the occurrence and development of HFSR, mainly in the EGFR signaling pathway, PI3K-Akt signaling pathway, RAS signaling pathway, HIF-1 signaling pathway, and FOXO signaling pathway. PPI network topology analysis showed that NTRK1, EGFR, APP, TP53, HSP90AB1, HSP90AA, and CUL3 were the core targets. According to the above analysis results, it was speculated that the treatment of HFSR might be through the following ways. At present, the more mature targeted drugs epidermal growth factor receptor inhibitors (EGFRIs) target epidermal growth factor receptor (EGFR), such as epidermal growth factor receptor tyrosine kinase inhibitors, which has been widely used in patients with advanced non-small cell lung cancer.^[31]^ EGFR is expressed in skin keratinocytes, sebaceous glands and hair follicle epithelial cells,^[32]^ and EGFRIs can also affect the epidermal growth factor signaling pathway of skin follicular stromal cells, increase cell differentiation, and induce inflammatory responses while anti-tumor causes skin toxicity.^[33]^ In addition, EGFRIs can also inhibit the expression of P13K-AKT, MAPK pathways and signaling molecules, thereby interfering with the differentiation function of keratinocytes, affecting the secretion of sebaceous glands, destroying the skin surface, reducing the ability of the epidermis to retain water, leading to the recruitment of inflammatory cells and skin damage, and eventually causing skin toxicity.^[34,35]^ Tumor-targeted drugs tend to accumulate in the sweat gland-rich parts of the palms and soles of the feet, resulting in excessive local drug concentrations, activation of the HIF-1 signaling pathway, hypoxia and necrosis of local tissue cells, resulting in adverse reactions of the hands and feet.^[36]^ Previous studies have shown that the active ingredients of Spatholobi Caulis can inhibit the above proteins and signaling pathways, providing a reliable basis for its mechanism of treating HFSR.^[37]^ Therefore, according to the results of this study, Spatholobi Caulis can effectively act on HFSR for the purpose of curing and preventing diseases.

In this study, computer simulation molecular docking technology was used to prove that Medicagol, the active ingredient in Spatholobi Caulis, had the highest affinity with EGFR, and the 2 formed a stable structure mainly through hydrogen bonding and hydrophobic interactions, thereby inactivating the enzyme. Studies have shown that Medicagol, a natural flavonoid compound, could exert anticancer effects by targeting EGFR-related signaling pathways.^[38]^ In addition, Medicagol encapsulated by nanofibers could enhance the proliferation of dermal fibroblasts and epidermal keratinocytes in vitro, and accelerate the epithelial regeneration and granulation tissue formation of superficial skin in vivo.^[39]^ These findings suggest that Medicagol may improve HFSR by inhibiting EGFR. And yet, there are still some deficiencies in this study, and further experimental studies need to be completed to confirm the results. Furthermore, interactions between active ingredients have not been considered.

## 5. Conclusions

In summary, the results of this study are consistent with previous studies, and the treatment of HFSR by Spatholobi Caulis has the characteristics of multi-target and multi-way regulation. In this study, the active components of Spatholobi Caulis were studied by the method of network pharmacology, and the potential targets and signaling pathways of HFSR were analyzed. At the same time, the most stable Medicagol binding to EGFR molecules was obtained through computer-simulated molecular docking experiments, which can be studied as a natural compound EGFR inhibitor. This study suggests that Medicagol may improve HFSR by inhibiting EGFR, providing a theoretical basis for the clinical treatment of HFSR.

## Acknowledgments

The authors would like to thank all the researchers in our working group.

## Author contributions

**Conceptualization:** Quan-yao Li, Huan-nan Meng.

**Data curation:** Quan-yao Li, Hui Liu.

**Formal analysis:** Li-qiu Yao.

**Funding acquisition:** He-xin Tan, Jun Shi.

**Investigation:** Huan-nan Meng, Hui Liu, He-xin Tan.

**Methodology:** Li-qiu Yao.

**Project administration:** He-xin Tan, Dan Lin, Jun Shi.

**Resources:** Dan Lin, Jun Shi.

**Software:** Quan-yao Li, Huan-nan Meng, Li-qiu Yao, Hui Liu.

**Validation:** Dan Lin, Jun Shi.

**Visualization:** Quan-yao Li, Li-qiu Yao, Hui Liu.

**Writing – original draft:** Quan-yao Li, Huan-nan Meng.

**Writing – review & editing:** Dan Lin, Jun Shi.
